# Lithium Response in Bipolar Disorder is Associated with Focal Adhesion and PI3K-Akt Networks: A Multi-omics Replication Study

**DOI:** 10.21203/rs.3.rs-3258813/v1

**Published:** 2023-10-03

**Authors:** John Kelsoe, Anna Ou, Sara Rosenthal, Mazda Adli, Kazufumi Akiyama, Nirmala Akula, Martin Alda, Azmeraw T. Amare, Raffaella Ardau, Bárbara Arias, Jean-Michel Aubry, Lena Backlund, Claudio Banzato, Michael Bauer, Bernhard Baune, Frank Bellivier, Antonio Benabarre, Susanne Bengesser, Bhattacharjee Abesh, Joanna Biernacka, Elise Bui, Pablo Cervantes, Guo-Bo Chen, Hsi-Chung Chen, Caterina Chillotti, Sven Cichon, Scott Clark, Francesc Colom, David Cousins, Cristiana Cruceanu, Piotr Czerski, Clarissa Dantas, Alexandre Dayer, Franziska Degenhardt, J. Raymond DePaulo, Bruno Etain, Peter Falkai, Frederike Fellendorf, Ewa Ferensztajn-Rochowiak, Andreas J. Forstner, Louise Frisen, Mark Frye, Janice Fullerton, Sebastien Gard, Julie Garnham, Fernando Goes, Maria Grigoroiu-Serbanescu, Paul Grof, Oliver Gruber, Ryota Hashimoto, Joanna Hauser, Urs Heilbronner, Stefan Herms, Per Hoffmann, Andrea Hofmann, Liping Hou, Stéphane Jamain, Esther Jiménez, Jean-Pierre Kahn, Layla Kassem, Tadafumi Kato, Sarah Kittel-Schneider, Barbara König, Po-Hsiu kuo, Ichiro Kusumi, Nina Dalkner, Gonzalo Laje, Mikael Landén, Catharina Lavebratt, Marion Leboyer, Susan Leckband, Carlos López Jaramillo, Glenda MacQueen, Mario Maj, Mirko Manchia, Cynthia Marie-Claire, Lina Martinsson, Manuel Mattheisen, Michael McCarthy, Susan McElroy, Francis McMahon, Philip Mitchell, Marina Mitjans, Francis Mondimore, Palmiero Monteleone, Caroline Nievergelt, Markus Nöthen, Tomas Novak, Urban Osby, Norio Ozaki, Sergi Papiol, Roy Perlis, Andrea Pfennig, James Potash, Daniela Reich-Erkelenz, Andreas Reif, Eva Reininghaus, Marcella Rietschel, Guy Rouleau, Janusz K. Rybakowski, Martin Schalling, Peter Schofield, Klaus Oliver Schubert, Thomas Schulze, BARBARA SCHWEIZER, Florian Seemüller, Giovanni Severino, Tatyana Shekhtman, Paul Shilling, Kazutaka Shimoda, Christian Simhandl, claire slaney, Alessio Squassina, Thomas Stamm, Pavla Stopkova, Sarah Tighe, Alfonso Tortorella, Gustavo Turecki, Eduard Vieta, Julia Volkert, Stephanie Witt, Naomi Wray, Adam Wright, Trevor Young, Peter Zandi, Maria Del Zompo

**Affiliations:** University of California San Diego; University of California San Diego; Department of Biological Psychiatry and Neuroscience, Dokkyo Medical University; National Institutes of Health, US Dept of Health & Human Services; Dalhousie University; University of Adelaide, AUSTRALIA; Hospital University Agency of Cagliari; Facultat de Biologia and Institut de Biomedicina (IBUB), Universitat de Barcelona, CIBERSAM; Geneva University Hospitals; University Hospital Carl Gustav Carus; University of Münster; Mayo Clinic; McGill University Health Centre; 3Department of Psychiatry, National Taiwan University Hospital, Taipei, Taiwan 4Department of Psychiatry, Center of Sleep Disorders, National Taiwan University Hospital, Taipei, Taiwan; Mayo Clinic; University of Adelaide; Max Plank Institute for Psychiatry; Poznan University of Medical Sciences; University of Geneva; University of Essen; Johns Hopkins University; University Hospital LMU; University of Bonn, School of Medicine & University Hospital Bonn; School of Medicine & University Hospital Bonn; Mayo Clinic; Neuroscience Research Australia; Alexandru Obregia Clinical Psychiatric Hospital; National Center of Neurology and Psychiatry; Institute of Psychiatric Phenomics and Genomics, University Hospital, LMU Munich; Institute of Human Genetics; National Institute of Mental Health Intramural Research Program, National Institutes of Health; Univ Paris Est Creteil, INSERM, IMRB; School of Medicine & University Hospital Bonn; Juntendo University Graduate School of Medicine; College of Public Health, National Taiwan University, Taipei, Taiwan; Hokkaido University Graduate School of Medicine; Gothenburg University; Karolinska Institutet; University of Naples, Italy; Dalhousie University; INSERM UMR-S 1144; Karolinska Institutet; Johns Hopkins University; Lindner Center; National Institute of Mental Health Intramural Research Program; National Institutes of Health; University of New South Wales; Max Planck Institute of Experimental Medicine, Göttingen, Germany; Neuroscience Research Australia; University of Salerno, University of Naples SUN; University of California, San Diego; School of Medicine & University Hospital Bonn; National Institute of Mental Health, Klecany; Karolinska Institutet; Nagoya University; University Hospital LMU; Massachusetts General Hospital; University Hospital Carl Gustav Carus, TU Dresden; University Hospital Frankfurt, Germany; University of Mannheim; McGill University; Poznan University of Medical Sciences; Karolinska Institutet; Neuroscience Research Australia; University of Adelaide; University of Munich; University of Cagliari; Universita degli Studi Di Cagliari; Charité - Universitätsmedizin Berlin, Campus Charité Mitte; Department of Psychiatry, University of Perugia, Perugia, Italy; Douglas Institute, Department of Psychiatry, McGill University; Hospital Clinic of Barcelona; University Medical Centre Mannheim; University of Queensland; Johns Hopkins University; University of Cagliari

## Abstract

Lithium is the gold standard treatment for bipolar disorder (BD). However, its mechanism of action is incompletely understood, and prediction of treatment outcomes is limited. In our previous multi-omics study of the Pharmacogenomics of Bipolar Disorder (PGBD) sample combining transcriptomic and genomic data, we found that focal adhesion, the extracellular matrix (ECM), and PI3K-Akt signaling networks were associated with response to lithium. In this study, we replicated the results of our previous study using network propagation methods in a genome-wide association study of an independent sample of 2,039 patients from the International Consortium on Lithium Genetics (ConLiGen) study. We identified functional enrichment in focal adhesion and PI3K-Akt pathways, but we did not find an association with the ECM pathway. Our results suggest that deficits in the neuronal growth cone and PI3K-Akt signaling, but not in ECM proteins, may influence response to lithium in BD.

## INTRODUCTION

Bipolar disorder (BD) is a chronic psychiatric illness that presents with episodes of mania, depression, and sometimes psychosis. Globally, it is the sixth leading cause of medical disability among people from 15 to 44 years old. Patients with BD are at a higher risk of suicide than those with any other psychiatric or medical illness. Some studies report that roughly 50% of patients will attempt suicide, and up to 20% of untreated patients will complete suicide^1^, while treatment by lithium reduces that risk significantly^2,3^.Unfortunately, misdiagnosis is common and often delays an accurate treatment. Up to 70% of patients are initially misdiagnosed, usually with major depressive disorder. On average, there is a delay of 8 years before the correct diagnosis of BD is made^4^. During this time, patients continue to suffer, may be treated with medications that make their illness course worse, and are at risk of suicide.

Lithium is the gold standard treatment for BD^5^. Its mechanism of action is still not completely understood^6^. Many studies have investigated the neurotrophic effect of lithium. One theory posits that chronic administration of lithium inhibits glycogen synthase kinase 3 (GSK3β), a serine/threonine kinase.This leads to anti-apoptotic effects and improved cell structural stability^7–10^. GSK3β has also been shown to exhibit interactions with many pathways, including phosphorylation of several components of the PI3K/AKT/mTOR signaling network, as well as regulation of transcription for proteins bound to microtubules^11^. Another theory involves the phosphoinositol (PI) cycle. In the PI cycle, lithium inhibits inositol monophosphatase, which ultimately downregulates protein kinase C isozymes such as myristoylated alanine-rich C-kinase substrate (MARCKS). MARCKS is an actin-binding protein found in neuronal processes that is implicated in cytoskeletal restructuring. Its downregulation stabilizes the neuronal membrane and results in neurotrophic effects^7,12^. A more recent theory proposes that lithium alters the phosphorylation state of collapsin response mediator protein-2 (CRMP2). CRMP2 regulates cytoskeletal organization, particularly in dendritic spines^13,14^. Finally, a study using polygenic score modeling has indicated that the cholinergic and glutamatergic pathways may potentially serve as targets for lithium^15^. It is possible that lithium exerts its effects through multiple or all of these pathways. A single definitive model remains elusive, but interactions with neuronal cytoskeleton are possibly involved.

Interestingly, there is a range of responses to treatment with lithium. Previous studies have reported that 20–30% of patients with BD are excellent responders, whereas over 40% fail to demonstrate any significant clinical improvement. These patient populations have been shown to differ from each other both phenotypically and genetically^16^. A differential response to lithium has been previously demonstrated between induced pluripotent stem cell (iPSC) neurons derived from lithium responders and non-responders. The hyperexcitability of in vitro neurons derived from BD patients was reversed by lithium treatment, but only in those from patients who were lithium responders^17^. This finding is also supported by family studies, which found that the relatives of lithium responders were significantly more likely to be lithium responders as well^18,19^. These studies imply that patients with BD could be subcategorized based on biological differences which induce a divergent lithium response. There is a great need to better understand these differences in order to identify possible predictors of treatment response. However, dozens of previous candidate-gene association studies, genome-wide association studies (GWAS), and polygenic risk score analyses of lithium response in BD have failed to identify genetic variants with major effects. Given this pressing need to find pharmacogenetic predictors of response, more advanced methods in integrative genomic analysis are necessary^16^.

GWAS inherently face several limitations when used in isolation, including the challenge of genetic heterogeneity. In many disease processes with genetic associations, patients may carry diverse combinations of causal variants that impact multiple genes, creating a net effect across a particular pathway. GWAS of BD primarily detect variants of very small effect size consistent with a polygenic mode of transmission. Since each single nucleotide polymorphism (SNP) contributes only a tiny amount to the overall predisposition to BD, enormous sample sizes are required, and it can be difficult to surmise mechanisms of disease. Network approaches seek to address this biological reality by integrating GWAS results with known protein-protein interactions and other molecular networks. New causal genes may be identi ed by boosting their interactions with products of known causal genes^20,21^.

We have recently reported a combined analysis of transcriptomic and GWAS data from the Pharmacogenomics of Bipolar Disorder (PGBD) study^22^ of treatment response to lithium. After using network propagation to reprioritize candidate genes from GWAS data, we found significant overlap between both transcriptomic and GWAS results. The joint analysis yielded a 500 gene network significantly enriched in the following Kyoto Encyclopedia of Genes and Genomes (KEGG) pathways: focal adhesion, ECM-receptor interaction, and PI3K-Akt signaling^23^. All three pathways play a role in axon growth and neuronal development^24^. Consistent with these results, post-mortem studies have found that in BD, neuronal populations may exhibit a decrease in number, size, and/or amount of dendritic spines^13,25^. Given that lithium may have downstream effects on these pathways, it is possible that genetic defects in focal adhesion pathways may provide both a mechanism for susceptibility to BD as well as a target for lithium treatment.

In this study, we aimed to replicate the results of our previous multi-omics study on a larger dataset of over 2,000 patients from the International Consortium on Lithium Genetics (ConLiGen)^26^. We reprioritized GWAS results using network methods to determine overlap with focal adhesion, ECM-receptor interaction, and PI3K-Akt signaling pathways.

## METHODS

Summary statistics were downloaded from the NHGRI-EBI GWAS Catalog^27^ on 12/12/2022 for study GCST012487^26^. The data resulted from a GWAS of lithium response in 2,563 patients at 22 sites participating in the International Consortium on Lithium Genetics (ConLiGen). We utilized the summary statistics from a combined sample of 2,039 European ancestry individuals. In the ConLiGen study, data from over 6 million single nucleotide polymorphisms (SNPs) were tested for association with categorical and continuous retrospective ratings of lithium response using the Alda scale^28,29^. The Alda scale includes two scores: score A is a 0–10 retrospective rating of lifetime response, while score B captures factors reducing the confidence in score A such as lack of a documented lithium level, etc. In the ConLiGen study, under the continuous phenotype, participants were rated with the Alda A score, and individuals with a B score greater than 4 were excluded. We used the continuous rather than the dichotomous phenotype as a measure of treatment response because genome-wide significant association was detected with the continuous phenotype in the original GWAS. Quality control and statistical analysis methods are described in the original paper.

### SNP, Gene, and Gene-Set Analysis

We imported the ConLiGen summary statistics into FUMA (Functional Mapping and Annotation of Genome-Wide Association Studies - https://fuma.ctglab.nl)^30^, a web-based platform for annotating, prioritizing, visualizing and interpreting GWAS results. We utilized the SNP2GENE function to map SNPs to genes and conduct SNP, gene-based, and gene-set analysis. We used all default settings, except for setting the maximum lead SNP p-value to 1×10e-5.

### Network Analysis

We input the ConLiGen summary statistics into NAGA (Network Assisted Genomic Analysis), an online network propagation tool for pathway boosting and interpretation of genome-wide association studies^21^.NAGA provided a reprioritized ranked list of 19,781 genes as output. We then entered the top 500 genes with the highest final heat scores into STRING, an online database that generates mapped networks based on protein-protein interactions^31^. STRING additionally analyzes for overrepresentation of user-inputted gene lists in established pathways, using the hypergeometric test^32^. Using this function, we tested our a priori hypotheses to identify functional enrichment of the NAGA-generated top 500 gene list in the KEGG hsa04510 focal adhesion pathway, KEGG hsa04512 ECM-receptor interaction, and KEGG hsa04151 PI3K-Akt signaling pathway^33^. P-values were corrected for multiple testing by STRING using the Benjamini–Hochberg procedure^34^.

Overlap between the NAGA-generated top 500 gene list and the KEGG pathways was visualized using Cytoscape^35^. A hypergeometric test was conducted to test for overrepresentation of the NAGA-generated 500 gene network in the 500 gene network generated in our previous study^23^.

## RESULTS

### Demographics

The demographics of the sample can be found in the original ConLiGen study^26^. The study was conducted in two phases: GWAS 1 (n = 1065) and GWAS 2 (n = 1168). Sex and age were similar across both cohorts. Mean Alda scale A scores were 6.13 (SD = 3.13) and 6.52 (SD = 2.87), respectively. Mean Alda scale B scores were 1.78 (SD = 1.26) and 2.35 (SD = 1.16), respectively.

### SNP, Gene, and Gene-Set Analysis

As reported in the original ConLiGen study, the only SNPs that were significant at a genome-wide significance level of 5e-08 were in linkage disequilibrium with the SNP rs74795342 on chromosome 21 (Supplemental Fig. 1). Using FUMA in our gene-wise analysis, no significant genes were found at a significance level of p < 0.05/18314 = 2.730e-6 (Supplemental Fig. 2). No gene-sets were found to be significant either, using p < 0.05 after Bonferonni correction. The most highly associated genes and gene-sets are listed in Supplemental Table 1 and Supplemental Table 2.

### Network Analysis

We first tested the three a priori pathways that were significant in our previous study, which had examined an independent sample^23^. Using the STRING analysis function, the top 500 reprioritized gene list generated by NAGA was found to be significantly enriched in both the KEGG hsa04510 focal adhesion pathway (*p* = 1.74e-06) and KEGG hsa04151 PI3K-Akt signaling pathway (*p* = 1.90e-07). Given the goal of replication and the small number of statistical tests, this was considered as a significant replication of our previous results in an independent sample for the focal adhesion and PI3K-Akt pathways. However, the KEGG hsa04512 ECM-receptor interaction pathway was not found to be significantly enriched.

A hypergeometric test found significant overlap (p = 5.699e-07) between the 500 gene network generated by NAGA and the 500 gene network generated by network propagation analysis in our previous study^23^.There were 33 genes that were common to both networks.

The top 25 reprioritized genes produced by NAGA are listed in Table 1. All top 500 reprioritized NAGA genes are listed in Supplemental Table 4.

After testing the three a priori hypotheses based on previous results, we tested the top 500 NAGA gene list for enrichment in all pathways in STRING. The top 10 KEGG pathways found to be most strongly enriched are found in Supplemental Table 3. These include cancer and growth pathways (such as Pathways in Cancer, Estrogen Signaling Pathway, Ras Signaling Pathway) as well as the dopaminergic synapse pathway.

We additionally used the STRING analysis function to test for functional enrichment of the top 100, 200, 300, and 400 reprioritized gene lists generated by NAGA in all three a priori KEGG pathways. The results agreed with the primary analysis, since all gene lists were significantly enriched in the KEGG hsa04510 focal adhesion pathway and KEGG hsa04151 PI3K-Akt signaling pathways at a level of p < .05. Only the top 100 reprioritized gene list was found to be significantly enriched in the KEGG hsa04512 ECM-receptor interaction pathway (*p* = .0050) inconsistent with a robust result. (Supplemental Table 5).

## DISCUSSION

In this study, we attempted to replicate our previous results which were from an independent sample^23^. We used network methods via NAGA to reprioritize GWAS results from the ConLiGen study on lithium response and used STRING to test three a priori network hypotheses: KEGG focal adhesion, ECM-receptor interaction and PI3K-Akt signaling. Two of these three networks, KEGG focal adhesion and PI3K-Akt signaling, were enriched in our top 500 reprioritized genes. However, we did not find significant enrichment for the ECM-receptor interaction pathway in the 500 gene network. Besides this pathway, we were otherwise able to replicate the results of our previous paper in a larger, independent sample of patients with BD. We found highly significant overlap between the top 500 gene network generated by NAGA in this study and the 500 gene network generated in the previous study, providing further evidence for replication.

Focal adhesions are points of contact between cells and proteins in the ECM. The formation of cell-ECM adhesion structures is initiated by cell surface integrins and driven by local actin polymerization. These structures function to not only mediate cell attachment to ECM, but also mediate transmembrane signaling. Integrin-ECM ligand binding can induce a number of downstream changes affecting cell shape, growth, and proliferation^36^. In neurons, specifically, the actin cytoskeleton of growth cones interacts with the ECM to guide axon development and extension^24,37^.

We had originally hypothesized that genetic deficits in focal adhesion, ECM, and PI3K-Akt pathways may impair axonal growth in neurons and determine response to lithium. Though one integrin protein was included in our top 500 genes, in general ECM proteins did not overlap with the top 500 gene list (Supplemental Table 4)(Fig. 2), and the pathway was not significant. This result is inconsistent with our previous study. However, it may suggest the possibility that the deficits influencing lithium response may be inherent to the growth cone rather than components of the ECM. This is supported by a number of studies, which have shown that lithium prevents collapse and induces growth of growth cones^38–40^.

Previously, neurons derived from induced pluripotent stem cells of patients with BD have been shown to exhibit hyperexcitability in vitro. This hyperexcitable phenotype was rescued by lithium only in neurons derived from lithium good responders^17^. Elevated neuroactivity in BD may induce vulnerability in neurons through impairment of focal adhesion pathways. Chronic elevation of neuroactivity has been shown to dramatically reduce surface expression of integrin β1 in animal models, leading to axonal and dendritic degeneration and eventually cell death^41^.

Unsurprisingly, neurons in patients with BD have been shown to be present with smaller size, fewer numbers, and more limited branching. We had previously proposed that in lithium responders, this deficit is caused by deficits in focal adhesion and is rescued by lithium treatment. Furthermore, we proposed that in patients who are not lithium responders, focal adhesion is not dysregulated, and lithium is unable to address the relevant impairments^42–44^. Our results in this study are consistent with this hypothesis.

**Figure 1:**
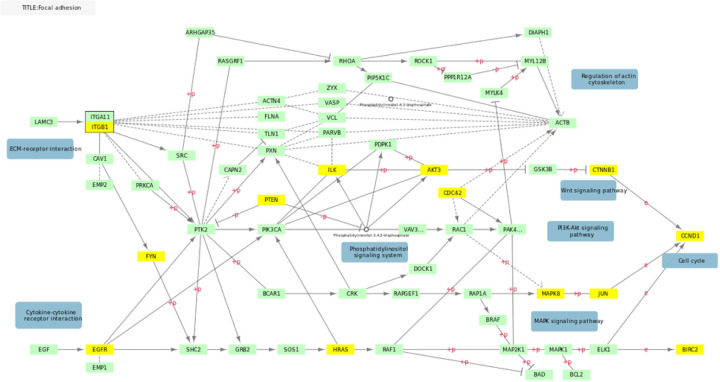
Overlap Between KEGG Focal adhesion and Top 500 Genes KEGG hsa04510 pathway for focal adhesion adapted to illustrate gene overlap. Genes in yellow overlap with the 500 gene NAGA network.

**Figure 2:**
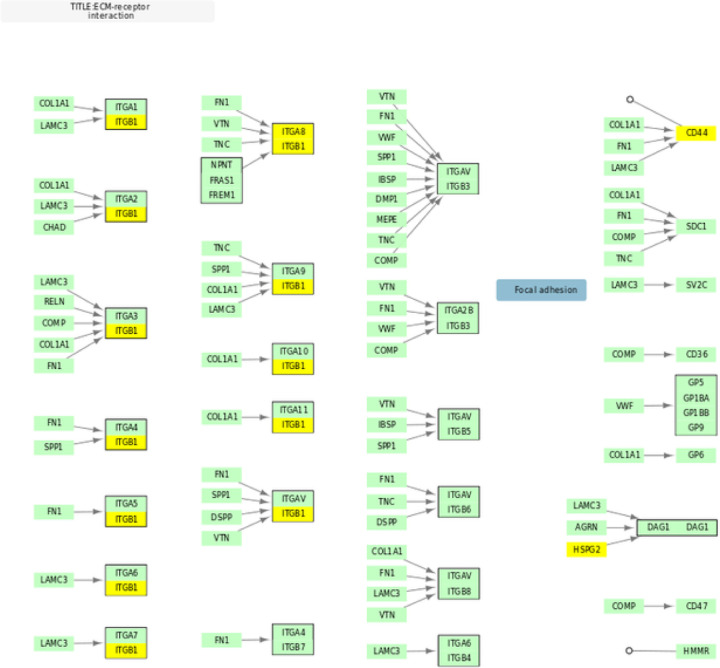
Overlap Between KEGG ECM-receptor interaction and Top 500 Genes KEGG hsa04512 pathway for ECM-receptor interaction adapted to illustrate gene overlap. Genes in yellow overlap with the 500 gene NAGA network.

After testing our three a priori hypotheses, we conducted exploratory analyses using network methods. We listed the top 10 most significant KEGG pathways that were associated by STRING with the NAGA generated gene-list in Supplemental Table 3. These pathways are mostly cancer pathways associated with cell growth and proliferation or pathways of addiction and other dopamine-related processes.Dopamine neurotransmission has previously been associated with response to lithium treatment in BD^45^.Genes in associated cancer pathways show some overlap with focal adhesion as well, which suggests the possibility of shared mechanisms. (Fig. 1).

Limitations of our study include the relatively small sample size (N = 2,039) and the generalizability of the data-set, given that all participants were of European descent. Additionally, data was collected retrospectively. As a result, outcomes may be less accurate in determining response phenotypes^46^ which can blur our findings due to false negatives.

This study also demonstrates the utility of network propagation methods, which can add power to GWAS with limited sample sizes. These methods are beneficial in identifying which genes and gene-sets are of interest to a disease process, but future research is still indicated for con rmation^20,21^.

In summary, we replicated our previous results reinforcing that genetic deficits in focal adhesion and PI3K-Akt signaling are associated with lithium response in BD patients. We hypothesize, as before, that malformed axonal growth cones result in shorter and less branched axons and susceptibility to BD in a subpopulation of patients who are lithium responders. This is also consistent with the idea that response to lithium results from a disease mechanism distinct from that of lithium non-responders. Furthermore, we propose that lithium rescues disrupted neuronal growth and axon extension processes by addressing deficits in focal adhesion. A better understanding of the pathophysiology of BD and lithium treatment may lead to the future development of drugs similar to lithium, as well as possible clinical predictors for treatment response.

## Figures and Tables

**Figure 3:**
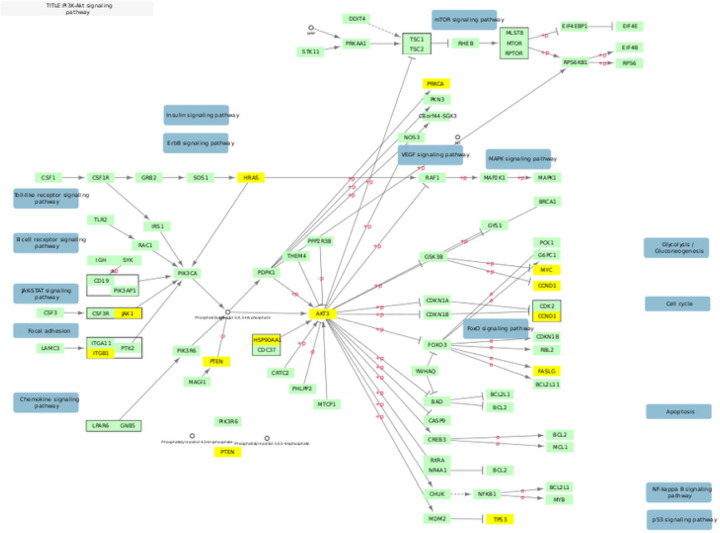
Overlap Between KEGG PI3k-Akt and Top 500 Genes KEGG hsa04151 pathway for PI3k-Akt signaling adapted to illustrate gene overlap. Genes in yellow overlap with the 500 gene NAGA network.

**Table 1:** NAGA Top 25 Gene List

NAGA				FUMA Gene-wise Analysis	
Gene	Input Heat	Final Heat	Rank	Rank	P-value
UBC	0	37.88310418	1	4335	.22849
GNB1	2.624306658	21.36254772	2	12993	.69873
PRKACB	6.770605441	19.12717575	3	5778	.30963
GNAL	0	18.71021909	4	16294	.88169
GNGT1	0	18.61047924	5	12956	.69712
REEP1	0	17.83396241	6	13325	.71728
ARRB1	7.336233161	17.55148794	7	12430	.66845
RTP2	0	17.50819164	8	11202	.60084
RTP1	0	17.50727661	9	13284	.71484
PRKACA	0	15.7274719	10	5506	.29380
ARRB2	0	15.43877781	11	14216	.76615
PRKACG	0	15.38554785	12	10721	.57518
GRK2	0	15.30495263	13	*	*
GNG13	0	14.28098978	14	9444	.50737
GNG7	0	14.26968321	15	2636	.13696
GRK3	0	13.36097413	16	*	*
TAF1	0	10.51679188	17	*	*
APP	0	9.571290241	18	11903	.63889
JUN	5.596453897	9.123060759	19	6091	.32547
HNF4A	0	7.372223527	20	13583	.73157
ELAVL1	0	7.080313649	21	2194	.1154
C1orf94	14.45796462	7.001838181	22	9155	.49203
CSMD2	14.45796462	6.383962772	23	36	.0016402
KCNJ5	11.85176049	6.256678752	24	13972	.75419
INS	3.272540349	5.764244643	25	10662	.57147

* = Data does not exist as gene was not evaluated in FUMA

**Table 2:** Functional Enrichment of NAGA Top 500 Gene List in Focal Adhesion, ECM, and PI3K Akt Pathways

Pathway	P-value	Number of genes overlapped
KEGG Focal Adhesion	1.74e-06*	21 of 198
KEGG ECM-receptor interaction	.1494	5 of 88
KEGG PI3k-Akt	1.90e-07*	31 of 350

All p-values corrected for multiple testing using the Benjamini–Hochberg procedure.

* = Significant at *p* < .05

## References

[1] Dome P., Rihmer Z. & Gonda X. Suicide Risk in Bipolar Disorder: A Brief Review. Medicina 55, (2019). 10.3390/medicina55080403 PMC6723289 31344941

[2] Cipriani A., Hawton K., Stockton S. & Geddes J. R. Lithium in the prevention of suicide in mood disorders: updated systematic review and meta-analysis. BMJ 346, f3646 (2013). 23814104 10.1136/bmj.f3646

[3] Plans L. Association between completed suicide and bipolar disorder: A systematic review of the literature. J. Affect. Disord. 242, 111–122 (2019). 30173059 10.1016/j.jad.2018.08.054

[4] Sajatovic M. Bipolar disorder: disease burden. Am. J. Manag. Care 11, S80–4 (2005). 16097718

[5] Rybakowski J. K. Lithium. Eur. Neuropsychopharmacol. 57, 86–87 (2022). 35189567 10.1016/j.euroneuro.2022.01.111

[6] Kato T. Mechanisms of action of anti-bipolar drugs. Eur. Neuropsychopharmacol. 59, 23–25 (2022). 35397446 10.1016/j.euroneuro.2022.03.008

[7] Machado-Vieira R., Manji H. K. & Zarate C. A. Jr. The role of lithium in the treatment of bipolar disorder: convergent evidence for neurotrophic effects as a unifying hypothesis. Bipolar Disord. 11 Suppl 2, 92–109 (2009). 19538689 10.1111/j.1399-5618.2009.00714.x PMC2800957

[8] Freland L. & Beaulieu J.-M. Inhibition of GSK3 by lithium, from single molecules to signaling networks. Front. Mol. Neurosci. 5, 14 (2012). 22363263 10.3389/fnmol.2012.00014 PMC3282483

[9] Jope R. S. Lithium and GSK-3: one inhibitor, two inhibitory actions, multiple outcomes. Trends Pharmacol. Sci. 24, 441–443 (2003). 12967765 10.1016/S0165-6147(03)00206-2

[10] Mishra H. K. Contributions of circadian clock genes to cell survival in broblast models of lithium-responsive bipolar disorder. Eur. Neuropsychopharmacol. 74, 1–14 (2023). 37126998 10.1016/j.euroneuro.2023.04.009 PMC11801370

[11] Hermida M. A., Dinesh Kumar J. & Leslie N. R. GSK3 and its interactions with the PI3K/AKT/mTOR signalling network. Adv. Biol. Regul. 65, 5–15 (2017). 28712664 10.1016/j.jbior.2017.06.003

[12] Watson D. G. & Lenox R. H. Chronic lithium-induced down-regulation of MARCKS in immortalized hippocampal cells: potentiation by muscarinic receptor activation. J. Neurochem. 67, 767–777 (1996). 8764606 10.1046/j.1471-4159.1996.67020767.x

[13] Tobe B. T. D. Probing the lithium-response pathway in hiPSCs implicates the phosphoregulatory set-point for a cytoskeletal modulator in bipolar pathogenesis. Proc. Natl. Acad. Sci. U. S. A. 114, E4462–E4471 (2017). 28500272 10.1073/pnas.1700111114 PMC5465887

[14] Zhao W.-N. Discovery of suppressors of CRMP2 phosphorylation reveals compounds that mimic the behavioral effects of lithium on amphetamine-induced hyperlocomotion. Transl. Psychiatry 10, 76 (2020). 32094324 10.1038/s41398-020-0753-6 PMC7039883

[15] Amare A. T. Association of polygenic score and the involvement of cholinergic and glutamatergic pathways with lithium treatment response in patients with bipolar disorder. Mol. Psychiatry (2023) doi:10.1038/s41380-023-02149-1. PMC11041653 37433967

[16] Papiol S., Schulze T. G. & Heilbronner U. Lithium response in bipolar disorder: Genetics, genomics, and beyond. Neurosci. Lett. 785, 136786 (2022). 35817312 10.1016/j.neulet.2022.136786

[17] Mertens J. Differential responses to lithium in hyperexcitable neurons from patients with bipolar disorder. Nature 527, 95–99 (2015). 26524527 10.1038/nature15526 PMC4742055

[18] Grof P. Is response to prophylactic lithium a familial trait? J. Clin. Psychiatry 63, 942–947 (2002). 12416605 10.4088/jcp.v63n1013

[19] Cruceanu C., Alda M. & Turecki G. Lithium: a key to the genetics of bipolar disorder. Genome Med. 1, 79 (2009). 19691823 10.1186/gm79 PMC2768965

[20] Leiserson M. D. M., Eldridge J. V., Ramachandran S. & Raphael B. J. Network analysis of GWAS data. Curr. Opin. Genet. Dev. 23, 602–610 (2013). 24287332 10.1016/j.gde.2013.09.003 PMC3867794

[21] Carlin D. E. A Fast and Flexible Framework for Network-Assisted Genomic Association. iScience 16, 155–161 (2019). 31174177 10.1016/j.isci.2019.05.025 PMC6554232

[22] Oedegaard K. J. The Pharmacogenomics of Bipolar Disorder study (PGBD): identi cation of genes for lithium response in a prospective sample. BMC Psychiatry 16, 129 (2016). 27150464 10.1186/s12888-016-0732-x PMC4857276

[23] Niemsiri V. Focal adhesion is associated with lithium response in bipolar disorder: evidence from a network-based multi-omics analysis. Mol. Psychiatry (2023) doi:10.1038/s41380-022-01909-9. PMC11078741 36991131

[24] Short C. A., Suarez-Zayas E. A. & Gomez T. M. Cell adhesion and invasion mechanisms that guide developing axons. Curr. Opin. Neurobiol. 39, 77–85 (2016). 27135389 10.1016/j.conb.2016.04.012 PMC4987159

[25] Maletic V. & Raison C. Integrated neurobiology of bipolar disorder. Front. Psychiatry 5, 98 (2014). 25202283 10.3389/fpsyt.2014.00098 PMC4142322

[26] Hou L. Genetic variants associated with response to lithium treatment in bipolar disorder: a genome-wide association study. Lancet 387, 1085–1093 (2016). 26806518 10.1016/S0140-6736(16)00143-4 PMC4814312

[27] Sollis E. The NHGRI-EBI GWAS Catalog: knowledgebase and deposition resource. Nucleic Acids Res. 51, D977–D985 (2023). 36350656 10.1093/nar/gkac1010 PMC9825413

[28] Manchia M. Assessment of Response to Lithium Maintenance Treatment in Bipolar Disorder: A Consortium on Lithium Genetics (ConLiGen) Report. PLoS One 8, e65636 (2013). 23840348 10.1371/journal.pone.0065636 PMC3686769

[29] Marie-Claire C., Courtin C., Bellivier F., Scott J. & Étain B. Methylomic Biomarkers of Lithium Response in Bipolar Disorder: A Proof of Transferability Study. Pharmaceuticals 15, (2022). 10.3390/ph15020133 PMC8877131 35215246

[30] Watanabe K., Taskesen E., van Bochoven A. & Posthuma D. Functional mapping and annotation of genetic associations with FUMA. Nat. Commun. 8, 1826 (2017). 29184056 10.1038/s41467-017-01261-5 PMC5705698

[31] Szklarczyk D. The STRING database in 2023: protein-protein association networks and functional enrichment analyses for any sequenced genome of interest. Nucleic Acids Res. 51, D638–D646 (2023). 36370105 10.1093/nar/gkac1000 PMC9825434

[32] Szklarczyk D. The STRING database in 2021: customizable protein-protein networks, and functional characterization of user-uploaded gene/measurement sets. Nucleic Acids Res. 49, D605–D612 (2021). 33237311 10.1093/nar/gkaa1074 PMC7779004

[33] Kanehisa M. & Goto S. KEGG: kyoto encyclopedia of genes and genomes. Nucleic Acids Res. 28, 27–30 (2000). 10592173 10.1093/nar/28.1.27 PMC102409

[34] Benjamini Y. & Hochberg Y. Controlling the false discovery rate: A practical and powerful approach to multiple testing. J. R. Stat. Soc. 57, 289–300 (1995).

[35] Shannon P. Cytoscape: a software environment for integrated models of biomolecular interaction networks. Genome Res. 13, 2498–2504 (2003). 14597658 10.1101/gr.1239303 PMC403769

[36] Wu C. Focal adhesion: a focal point in current cell biology and molecular medicine. Cell Adh. Migr. 1, 13–18 (2007). 19262093 10.4161/cam.1.1.4081 PMC2633675

[37] Omotade O. F., Pollitt S. L. & Zheng J. Q. Actin-based growth cone motility and guidance. Mol. Cell. Neurosci. 84, 4–10 (2017). 28268126 10.1016/j.mcn.2017.03.001 PMC5587356

[38] Williams R. S. B., Cheng L., Mudge A. W. & Harwood A. J. A common mechanism of action for three mood-stabilizing drugs. Nature 417, 292–295 (2002). 12015604 10.1038/417292a

[39] Shah S. M., Patel C. H., Feng A. S. & Kollmar R. Lithium alters the morphology of neurites regenerating from cultured adult spiral ganglion neurons. Hear. Res. 304, 137–144 (2013). 23856237 10.1016/j.heares.2013.07.001 PMC3773701

[40] Owen R. & Gordon-Weeks P. R. Inhibition of glycogen synthase kinase 3β in sensory neurons in culture alters lopodia dynamics and microtubule distribution in growth cones. Mol. Cell. Neurosci. 23, 626–637 (2003). 12932442 10.1016/s1044-7431(03)00095-2

[41] Murase S. Impaired Focal Adhesion Kinase-Grb2 Interaction during Elevated Activity in Hippocampal Neurons. Int. J. Mol. Sci. 16, 15659–15669 (2015). 26184168 10.3390/ijms160715659 PMC4519918

[42] Gigante A. D. Morphometric post-mortem studies in bipolar disorder: possible association with oxidative stress and apoptosis. Int. J. Neuropsychopharmacol. 14, 1075–1089 (2011). 21205433 10.1017/S146114571000146X

[43] Konradi C. Hippocampal interneurons in bipolar disorder. Arch. Gen. Psychiatry 68, 340–350 (2011). 21135314 10.1001/archgenpsychiatry.2010.175 PMC3197787

[44] Rajkowska G. Cell pathology in bipolar disorder. Bipolar Disord. 4, 105–116 (2002). 12071508 10.1034/j.1399-5618.2002.01149.x

[45] Mohamadian M. Mood and behavior regulation: interaction of lithium and dopaminergic system. Naunyn. Schmiedebergs. Arch. Pharmacol. (2023) doi:10.1007/s00210-023-02437-1. 36843130

[46] Talari K. & Goyal M. Retrospective studies - utility and caveats. J. R. Coll. Physicians Edinb. 50, 398–402 (2020). 33469615 10.4997/JRCPE.2020.409

